# Cerebral glucose metabolic prediction from amnestic mild cognitive impairment to Alzheimer’s dementia: a meta-analysis

**DOI:** 10.1186/s40035-018-0114-z

**Published:** 2018-04-23

**Authors:** Hai Rong Ma, Li Qin Sheng, Ping Lei Pan, Gen Di Wang, Rong Luo, Hai Cun Shi, Zhen Yu Dai, Jian Guo Zhong

**Affiliations:** 1 Department of Neurology, Traditional Chinese Medicine Hospital of Kunshan, Kunshan, People’s Republic of China; 2 Department of Neurology, School of Medicine, Affiliated Yancheng Hospital, Southeast University, West Xindu Road 2#, Yancheng, Jiangsu Province 224001 People’s Republic of China; 3 Department of Radiology, School of Medicine, Affiliated Yancheng Hospital, Southeast University, West Xindu Road 2#, Yancheng, Jiangsu Province 224001 People’s Republic of China

**Keywords:** Alzheimer’s dementia, Mild cognitive impairment, FDG-PET, Conversion, Meta-analysis, Seed-based *d* mapping

## Abstract

Brain ^18^F-fluorodeoxyglucose positron emission tomography (FDG-PET) has been utilized to monitor disease conversion from amnestic mild cognitive impairment (aMCI) to Alzheimer’s dementia (AD). However, the conversion patterns of FDG-PET metabolism across studies are not conclusive. We conducted a voxel-wise meta-analysis using Seed-based *d* Mapping that included 10 baseline voxel-wise FDG-PET comparisons between 93 aMCI converters and 129 aMCI non-converters from nine longitudinal studies. The most robust and reliable metabolic alterations that predicted conversion from aMCI to AD were localized in the left posterior cingulate cortex (PCC)/precuneus. Furthermore, meta-regression analyses indicated that baseline mean age and severity of cognitive impairment, and follow-up duration were significant moderators for metabolic alterations in aMCI converters. Our study revealed hypometabolism in the left PCC/precuneus as an early feature in the development of AD. This finding has important implications in understanding the neural substrates for AD conversion and could serve as a potential imaging biomarker for early detection of AD as well as for tracking disease progression at the predementia stage.

**Electronic supplementary material:**

The online version of this article (10.1186/s40035-018-0114-z) contains supplementary material, which is available to authorized users.

## Background

Alzheimer’s dementia (AD), an age-related neurodegenerative disorder, is the most common type of dementia worldwide, placing heavy burdens on global economic and social development [1–3]. Mild cognitive impairment (MCI), especially in its amnestic form, is considered a prodromal state of AD as it is associated with an annual conversion rate to AD of 10–15% [4]. However, a substantial proportion of patients with MCI will not progress to AD or other types of dementia, and may even remain stable or revert to normal cognition [5]. Individuals with amnestic MCI (aMCI) identified during prodromal phases as being at risk for conversion to AD may benefit most from early interventions. Unfortunately, it is difficult to differentiate aMCI converters from non-converters using clinical and neuropsychological assessments.

In contrast to brain amyloid-beta positron emission tomography (PET) that has no place in the routine work-up of suspected AD [6], brain ^18^F-fluorodeoxyglucose (FDG) PET is now a well-established tool in detecting and defining the distribution of neural injury or synaptic dysfunction in AD and MCI/aMCI [7–9]. Cerebral hypometabolism assessed with FDG-PET in AD patients was frequently observed in the posterior cingulate cortex (PCC), precuneus, and parietotemporal association areas [7, 10]. A similar distribution has also been identified in patients with MCI/aMCI, whereas the magnitude and spatial extent of hypometabolism is less than that observed in patients with AD [10, 11]. Many longitudinal studies have utilized FDG-PET to aid in predicting the conversion from aMCI to AD [12, 13]. However, the cerebral regions where early FDG-PET changes occur are not always concordant across studies. For example, when comparing aMCI converters to non-converters, glucose hypometabolism has been identified in a wide range of brain regions, including in the temporoparietal association areas [14–18], PCC [17–20], precuneus [21], medial and lateral temporal lobes [16, 18, 20, 22], medial and lateral frontal cortices (including the anterior cingulate cortex [ACC]) [17, 19, 20, 23]. Given the variability, Schroeter et al. preliminarily conducted a meta-analysis of voxel-based neuroimaging studies using anatomical likelihood estimation (ALE) and found that the left inferior parietal lobe and right precuneus were the most consistent regions that could predict the conversion from MCI to AD [24]. However, their results should be interpreted with caution as they were derived from the integration of studies using FDG-PET, single photon emission computed tomography (SPECT), and structural magnetic resonance imaging (MRI) modalities [24]. In addition, no FDG-PET studies after 2007 were included [14, 21], with several more FDG-PET studies on larger samples having been conducted since then [16, 17, 19, 20, 22, 23]. Furthermore, quantitative voxel-based meta-analytic methods have improved in recent years, allowing for a more accurate analysis [25–27]. Therefore, further investigation is necessary to verify the finding by Schroeter et al. that changes in the left inferior parietal lobe and right precuneus can predict the conversion from MCI to AD [24].

The aim of this work was to carry out a modified meta-analysis in order to identify brain regions of FDG-PET metabolic alterations that could be used to predict the conversion from aMCI to AD. In order to do this, we pooled voxel-based FDG-PET studies to determine the most consistent and reliable brain regions that showed alterations in glucose metabolism. The meta-analysis was conducted using Seed-based *d* Mapping (SDM), a popular meta-analytical technique [26, 28–31] that has been well-validated and employed to analyze voxel-based neuroimaging studies to explore the robustness of brain activity or structural changes in many neuropsychiatric disorders [27, 32–36].

## Methods

### Literature search and study selection

Electronic searches were conducted in the PubMed, Web of Science, and Embase databases using a combination of keywords (“Mild cognitive impairment” OR “Alzheimer’s disease”) AND ((“Positron Emission Tomography” OR “PET”) AND (“Fluorodeoxyglucose” OR “FDG”)) OR “FDG-PET”) on February 17, 2017. No publication language or date restrictions were used for the electronic searches. The reference lists of relevant original studies, systematic reviews, and meta-analyses were manually screened for additional qualified publications as well. Criteria for considering studies for this meta-analysis were as follows: (1) studies were published as original research articles in peer-reviewed journals; (2) studies were longitudinal that followed patients diagnosed with aMCI (single domain or multiple domains) [37–41] to determine which of them converted to AD; (3) studies conducted a direct voxel-based statistical comparison of baseline FDG-PET metabolic differences between patients with aMCI who converted to AD (aMCI converters) and those that did not (aMCI non-converters); (4) studies reported peak Montreal Neurological Institute (MNI) or Talairach/Tournoux coordinates; (5) significant results of regional FDG-PET metabolic differences were reported within one study using a consistent statistical threshold. In cases with multiple studies and overlapping patient groups that reported similar neuroimaging findings, the study with the largest sample size was included. Exclusion criteria were as follows: (1) studies enrolled subjects with aMCI that subsequently converted to other dementia types during the follow-up or enrolled patients with non-aMCI at baseline; (2) studies did not report peak coordinates or they were not obtained after contacting the corresponding author; (3) studies limited their analyses to ROI approaches only. Recorded data were extracted from each included study, including the first author’s name, year of publication, mean age, gender, and sample size of aMCI converters and non-converters, cognitive impairment severity (mean Mini-Mental State Examination (MMSE) score), education level, follow-up duration, diagnostic criteria, and imaging characteristics (e.g., post-processing software, smoothing kernel, and statistical threshold). In addition, peak coordinates and their effect sizes (e.g., t-values) of regional FDG-PET metabolic differences between aMCI converters and non-converters were extracted for the following voxel-wise meta-analysis. The literature search and selection, and data extraction were independently performed by two investigators (H.R.M. and L.Q.S). Any discrepancies were discussed with another investigator (J.G.Z.) until they were resolved by consensus. This work was conducted in accordance with the guidelines set by the Meta-analysis Of Observational Studies in Epidemiology (MOOSE) [42].

### Data analysis

#### Main voxel-wise meta-analysis

Regional glucose metabolism differences between aMCI converters and aMCI non-converters were meta-analyzed in a voxel-wise manner using the SDM software package (latest version 5.141, available at https://www.sdmproject.com/). The SDM approach has been described in detail in other publications [26, 28–30] and the tutorial is available for review (http://www.sdmproject.com/software/tutorial.pdf). Briefly, an effect-size signed map and an effect-size variance map from the peak coordinates and their effect sizes of regional glucose metabolic differences between aMCI converters and aMCI non-converters was first separately recreated for each individual FDG-PET study using an anisotropic via a 20 mm full-width at half maximum (FWHM) un-normalized Gaussian kernel. It should be noted that this kernel is designed to assign indicators of proximity to report coordinates but not to smooth any image, which is different in nature from the smoothing kernel. Moreover, this kernel has been found to have excellent control over false positives. Following this, a mean map was created by calculating the random-effects mean of the study maps in a voxel-wise manner, which was weighted by sample size and intra-study variability, and additional between-study heterogeneity. Statistical significance was determined using a default threshold (*p* = 0.005, peak height Z = 1, cluster extent = 10 voxels), which optimized the balance of false positives and negatives [26, 28].

#### Sensitivity analyses

In order to assess the replicability of the results, a whole-brain voxel-based jackknife sensitivity analysis was performed by iteratively repeating the same statistical analysis, removing one study at a time [28, 29]. Jackknife analyses were thresholded with the default settings (*p* = 0.005, peak height Z = 1, cluster extent = 10 voxels) [26, 28].

#### Heterogeneity analysis

A random-effects model with Q statistics (a chi-square distribution converted to z values) was utilized to examine the between-study heterogeneity of individual clusters. A permutation approach (p = 0.005, peak height z = 1, cluster extent = 10 voxels) was used to determine statistical significance [43].

#### Publication bias analysis

Possible publication biases in the main meta-analytic peaks were examined by analyzing the funnel plots and Egger’s tests [44]. Significance was determined as an asymmetry of funnel plots and a *p*-value less than 0.05 on Egger’s test.

#### Meta-regression analysis

Meta-regression analyses were conducted to explore the confounding effects of baseline mean age and severity of cognitive impairment (mean MMSE score), and follow-up duration that could potentially influence the meta-analytic results. Statistical significance was thresholded at a more conservative p-value of 0.0005 and a cluster extent of 10 voxels in accordance with previous meta-analyses [28, 30].

## Results

### Included studies

**Fig. 1:**
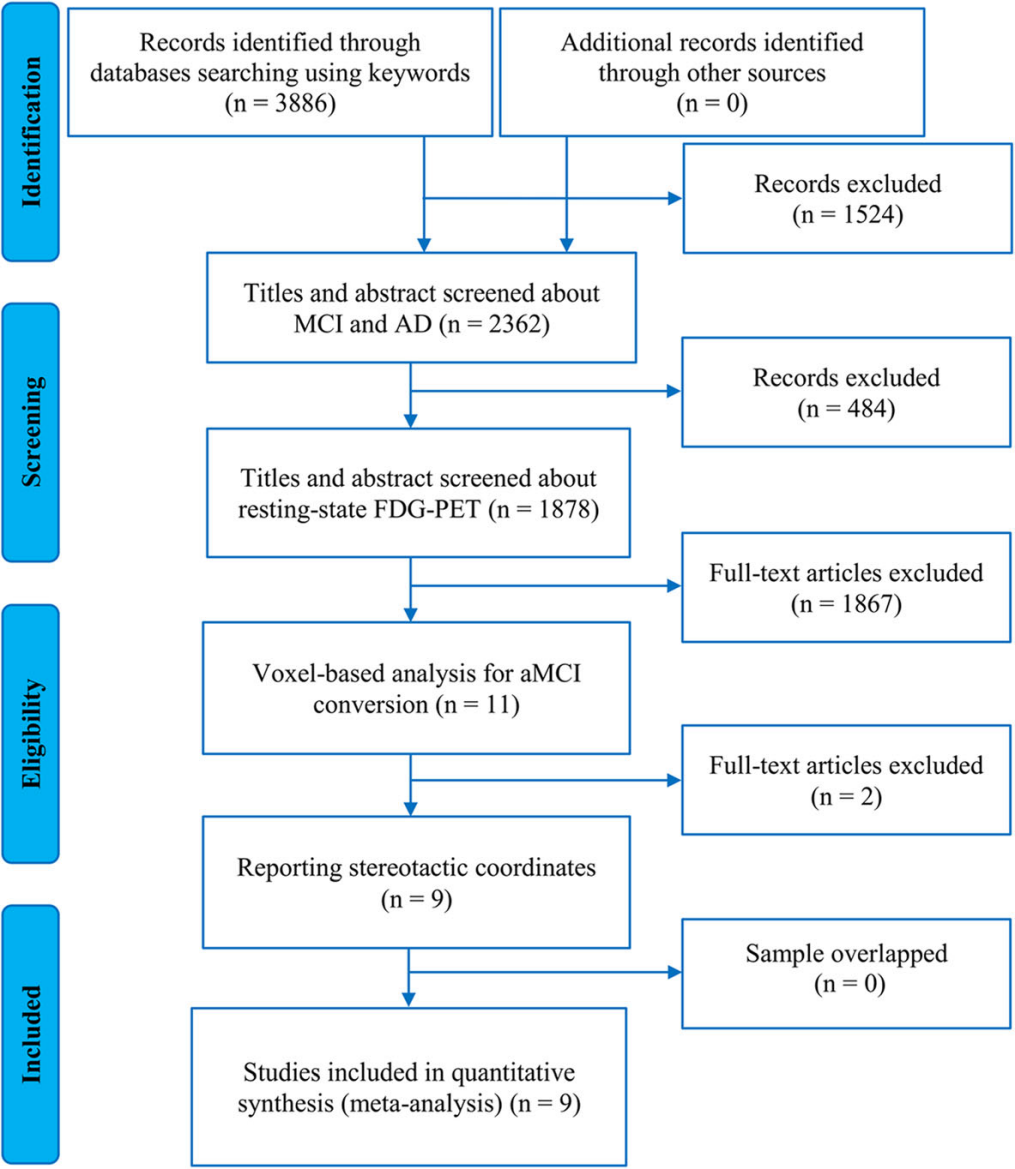
Flow chart for the literature search. Abbreviations: MCI, mild cognitive impairment; AD, Alzheimer’s dementia; FDG-PET, ^18^F-fluorodesoxyglucose positron emission tomography; aMCI, amnestic MCI

Our initial search yielded a total of 3886 titles reviewed for inclusion. After a careful screen of the literatures according to the inclusion and exclusion criteria, nine original studies that reported 10 comparisons [14–16, 19–23, 45] were finally eligible for the meta-analysis (Fig. 1), which included 93 patients classified as aMCI converters (mean age range 57.8–77.7 years and mean MMSE scores range 19.9–27.8) and 129 patients classified as aMCI non-converters (mean age range 60–75.8 years and mean MMSE scores range 23.9–28.3). The mean follow-up durations ranged from 12 to 60 months. Details of demographic, clinical and imaging characteristics of the included studies are summarized in Table 1.

**Table 1:** Characteristics of FDG-PET studies included in the meta-analysis

Study	Sample size (female)		Age (SD)	Education (years)	MMSE (SD)	Software	FWHM	Threshold	Follow-up Duration	Diagnostic Criteria
Chetelat et al., 2003 [14]	Converters	7 (4)	73 (5.1)	NA	26.3 (1)	SPM99	NA	0.05, corrected	18 months	Petersen et al. (2001) [37]
	Non-converters	10 (5)	67.8 (7)	NA	27.8 (1.2)					
Drzezga et al., 2003 [21]	Converters	8 (3)	71.6 (4.3)	NA	27.9 (1.2)	SPM99	12 mm	0.001, uncorrected	12 months	Petersen et al. (1999) [38]
	Non-converters	12 (7)	68.6 (6.5)	NA	28.1 (1.3)					
Mosconi et al., 2004 [15]	Converters	8 (NA)	71 (5)	8 (3)	26.7 (1.3)	SPM99	12 mm	0.05, corrected	12.0 months	Petersen et al. (2001) [37]
	Non-converters	29 (NA)	66 (8)	10 (5)	28.3 (1.4)				12.1 months	
Salmon et al., 2008 [19]	Converters	17 (12)	71.3 (5.9)	10.8 (2.5)	24.0 (2.2)	SPM2	12 mm	0.005, uncorrected	36 months	Petersen et al. (2001) [37] and Winblad et al. (2004) [39]
	Non-converters	17 (7)	66.2 (7.0)	12.3 (5.2)	25.7 (1.9)					
Fouquet et al., 2009 [23]	Converters	7 (5)	73.3 (4.3)	11.0 (4.7)	26.7 (1.0)	SPM2	10 mm	0.005, uncorrected	17.5 months	Petersen et al. (2001) [37]
	Non-converters	10 (6)	70.4 (11.2)	9.9 (3.6)	28.1 (1.0)				18.2 months	
Kim et al., 2010 [20]	Converters^a^	7 (NA)	57.8 (3.7)	NA	27.00 (1.15)	SPM2	12 mm	0.001, uncorrected	60 months	Petersen et al. (1999) [38]
	Non-converters^a^	5 (NA)	60.0 (4.2)	NA	28.00 (2.12)					
Kim et al., 2010 [20]	Converters^b^	8 (NA)	71.7 (4.4)	NA	27.50 (1.87)	SPM2	12 mm	0.001, uncorrected	60 months	Petersen et al. (1999) [38]
	Non-converters^b^	6 (NA)	75.8 (4.1)	NA	26.63 (1.69)					
Morbelli et al., 2010 [22]	Converters	9 (7)	77.1 (5.9)	8.6 (4.3)	27.8 (0.9)	SPM2	10 mm	0.005, uncorrected	22.6 months	Petersen et al. (2004) [40]
	Non-converters	11 (5)	74.0 (5.3)	8.7 (4.5)	27.8 (1.4)				26.5 months	
Pagani et al., 2010 [16]	Converters	10 (8)	77.7 (4.8)	8.8 (3.9)	27.5 (1.4)	SPM2	8 mm	0.05, corrected	22.0 months	Petersen et al. (2004) [40]
	Non-converters	9 (4)	75.8(5.9)	11.0 (5.3)	27.0 (2.0)				23.7 months	
Sohn et al., 2015 [45]	Converters	12 (9)	69.5 (7.7)	7.5 (2.9)	19.9 (3.5)	SPM8	12 mm	0.005, uncorrected	24 months	Albert et al. (2011) [41]
	Non-converters	20 (14)	71.6 (7.0)	9.2 (4.1)	23.9 (3.4)					

*Abbreviations*: *FDG-PET* ^18^F-fluorodesoxyglucose positron emission tomography, *SD* standard deviation, *MMSE* Mini-Mental State Examination, *FWHM* full width at half maximum, *NA* not available, *SPM* statistical parametric mapping

^a^early-onset amnestic mild cognitive impairment

^b^late-onset amnestic mild cognitive impairment

### Main voxel-wise meta-analysis

**Fig. 2:**
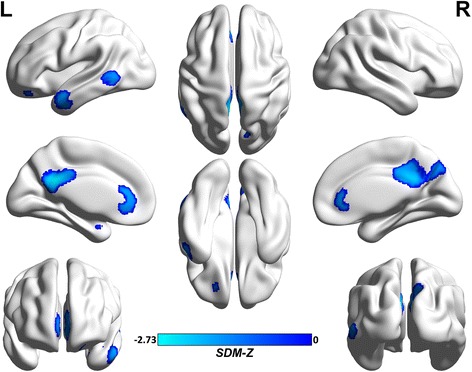
Brain hypometabolism map for the main voxel-wise meta-analysis in aMCI converters and aMCI non-converters. Abbreviations: aMCI, amnestic mild cognitive impairment; L, left; R, right; SDM, Seed-based *d* Mapping. The color bar indicates the maximum and the minimum SDM-Z values

As shown in Fig. 2 and Table 2, the main voxel-wise meta-analysis identified significant regional hypometabolism in the left/right PCC/precuneus (BAs 23, 7, and 30), left/right ACC (BAs 11, 32, 24, and 10), left middle temporal gyrus (BAs 21, 20, and 38), left middle temporal gyrus (BAs 21 and 37), and left middle frontal gyrus, orbital part (BA 11) in aMCI converters relative to aMCI non-converters at baseline.

**Table 2:** Clusters of regional hypometabolism in aMCI converters relative to aMCI non-converters

Anatomical label	Peak MNI coordinate (x, y, z)	Number of voxels	SDM-Z value	*p* value (SDM)	p value (Egger’s test)
Left/Right PCC/precuneus (BAs 23, 7, and 30)	−4, −48, 28	1959	−2.73	~ 0	0.37
Left/Right ACC (BAs 11, 32, 24, and 10)	−4, 32, 0	668	−1.72	0.00047	0.23
Left middle temporal gyrus (BAs 21, 20, and 38)	−44, 4, − 26	501	−1.66	0.00067	0.16
Left middle temporal gyrus (BAs 21 and 37)	−58, −52, −2	185	− 1.65	0.00074	0.13
Left middle frontal gyrus, orbital part (BA 11)	−20, 40, −18	22	−1.39	0.0031	0.30

*Abbreviations*: *MCI* amnestic mild cognitive impairment, *MNI* Montreal Neurological Institute, *SDM* Seed-based *d* Mapping, *PCC* posterior cingulate cortex, *ACC* anterior cingulate cortex, *BA* Brodmann area

### Sensitivity analyses

The jackknife sensitivity analyses revealed that regional hypometabolism in the left/right PCC/precuneus (BAs 23, 7, and 30) was the most reliable alteration as it was replicable in all 10 comparisons. Regions of hypometabolism in the left/right ACC (BAs 11, 32, 24, and 10) and left middle temporal gyrus (BAs 21, 20, and 38) were replicable in all but one comparison combinations. Regions of hypometabolism in the left middle temporal gyrus (BAs 21 and 37) and left middle frontal gyrus, orbital part (BA 11) failed to emerge in two of the comparison combinations. These results are summarized in Table 3.

**Table 3:** Jackknife sensitivity analysis

All studies but …	Left/Right PCC/precuneus (BAs 23, 7, and 30)	Left/Right ACC (BAs 11, 32, 24, and 10)	Left middle temporal gyrus (BAs 21, 20, and 38)	Left middle temporal gyrus (BAs 21 and 37)	Left middle frontal gyrus, orbital part (BA 11)
Chetelat et al., 2003 [14]	Yes	Yes	Yes	Yes	Yes
Drzezga et al., 2003 [21]	Yes	Yes	Yes	Yes	Yes
Mosconi et al., 2004 [15]	Yes	Yes	Yes	Yes	Yes
Salmon et al., 2008 [19]	Yes	No	Yes	No	No
Fouquet et al., 2009 [23]	Yes	Yes	Yes	Yes	Yes
Kim et al., 2010^a^ [20]	Yes	Yes	Yes	Yes	No
Kim et al., 2010^b^ [20]	Yes	Yes	Yes	Yes	Yes
Morbelli et al., 2010 [22]	Yes	Yes	No	Yes	No
Pagani et al., 2010 [16]	Yes	Yes	Yes	No	Yes
Sohn et al., 2015 [45]	Yes	Yes	Yes	Yes	Yes
Total	10 out of 10	9 out of 10	9 out of 10	8 out of 10	8 out of 10

*Abbreviations*: *PCC* posterior cingulate cortex, *ACC* anterior cingulate cortex, *BA* Brodmann area, *Yes* the region(s) reported, *No* the region(s) not reported

^a^early-onset amnestic mild cognitive impairment

^b^late-onset amnestic mild cognitive impairment

### Heterogeneity analysis

As demonstrated in Additional file 1: Figure S1 and Table 4, significant between-study heterogeneity was identified in the right PCC/precuneus (BA 23), right superior temporal gyrus (BAs 22 and 21), left middle frontal gyrus (BA 46), left/right ACC (BAs 11, 32, 24, and 25), left middle frontal gyrus, orbital part (BA 11), and left middle temporal gyrus (BAs 21 and 20).

**Table 4:** Regions with heterogeneity

Anatomical label	Peak MNI coordinate (x, y, z)	Number of voxels	SDM-Z value	*p* value
Right PCC/precuneus (BA 23)	12, −40, 34	374	1.49	0.00012
Right superior temporal gyrus (BAs 22 and 21)	60, −28, 6	440	1.20	0.00037
Left middle frontal gyrus (BA 46)	−28, 40, 30	270	1.57	0.000084
Left/Right ACC (BAs 11, 32, 24, and 25)	−4, 36, 4	216	1.25	0.00030
Left middle frontal gyrus, orbital part (BA 11)	−18, 58, −14	184	1.26	0.00029
Left middle temporal gyrus (BAs 21 and 20)	−50, 2, − 28	85	1.12	0.00056

*Abbreviations*: *SDM* Seed-based *d* Mapping, *MNI* Montreal Neurological Institute, *PCC* posterior cingulate cortex, *ACC* anterior cingulate cortex, *BA* Brodmann area

### Publication bias analysis

No significant publication biases were detected in the regions obtained from the main voxel-wise meta-analysis as revealed by the relative symmetry of the funnel plots (Additional file 2: Figure S2) and statistically non-significant Egger’s tests (Table 2).

### Meta-regression analysis

A meta-regression analysis revealed that aMCI-converters with an older mean age at baseline (available from all 10 comparisons) exhibited more regional hypometabolism in the left middle temporal gyrus (BAs 21, 20, and 37) and less regional hypometabolism in the left superior/middle frontal gyri, orbital part (BA 11). Lower mean MMSE scores of aMCI converters at baseline (available from all 10 comparisons) were associated with more regional hypometabolism in the left middle frontal gyrus (BA 9) and left temporal pole (BAs 28 and 36). In addition, findings of the meta-regression analysis suggested that a longer mean follow-up duration in aMCI-converters (available from all 10 studies) was associated with more regional hypometabolism in the left middle frontal gyrus (BAs 46 and 9). The results of these meta-regression analyses are summarized in Table 5.

**Table 5:** Meta-regression analyses

	Anatomical label	Peak MNI coordinate (x, y, z)	Number of voxels	SDM-Z value	*p* value
Effect of age	Association of regional glucose metabolism effect size and older mean age of aMCI converters				
	Left middle temporal gyrus (BAs 21 and 20)	−54, 4, − 28	152	−1.64	0.00024
	Left middle temporal gyrus (BA 37)	−56, −58, −2	64	−1.69	0.00016
	Left superior /middle frontal gyri, orbital part (BA 11)	−18, 58, − 14	53	1.73	0.000076
Effect of illness severity	Association of regional glucose metabolism effect size and lower mean MMSE scores of aMCI converters				
	Left middle frontal gyrus (BA 9)	−24, 22, 42	21	−2.74	0.00014
	Left temporal pole (BAs 28 and 36)	−20, 8, −34	13	−2.69	0.00017
Effect of follow-up duration	Association of regional glucose metabolism effect size and longer mean follow-up duration				
	Left middle frontal gyrus (BAs 46 and 9)	−30, 40, 30	255	−3.30	0.000037

*Abbreviations*: *SDM* Seed-based *d* Mapping, *MNI* Montreal Neurological Institute, *aMCI* amnestic mild cognitive impairment, *PCC* posterior cingulate cortex, *ACC* anterior cingulate cortex, *BA* Brodmann area

## Discussion

To the best of our knowledge, this was the first voxel-wise meta-analysis that only pooled FDG-PET studies to determine the most robust brain regions with glucose metabolism alterations in prediction conversion from aMCI to AD. In addition to the main voxel-wise meta-analysis in this study, several complementary analyses, such as jackknife sensitivity, heterogeneity, and publication bias analyses were conducted to test the robustness of our results. Pooling these findings, we observed that regional hypometabolism in the left PCC/precuneus at baseline was the most reliable and robust difference between aMCI converters and aMCI non-converters. Moreover, meta-regression analyses revealed that regional hypometabolism in the left PCC/precuneus in aMCI converters relative to aMCI non-converters at baseline was not biased by potential confounding variables, such as mean age and severity of global cognitive impairment at baseline, or follow-up durations across studies, which lend further support for the robustness of this finding.

The PCC and its adjacent precuneus, situated in the posteromedial part of the parietal cortex, make intensive connections with the medial temporal lobe (MTL) and other cerebral areas [46–48]. The PCC/precuneus are believed to play a pivotal role in the default mode network (DMN), which shows the highest level of baseline activity at rest and is deactivated during tasks in healthy subjects [49–51]. Impairments in the DMN, one of the most investigated intrinsic networks in patients with AD and aMCI, contribute to characteristic cognitive deficits, such as episodic memory and visuospatial processes [17, 46, 48, 52–55]. Compelling evidence from functional and metabolic studies suggests prominent involvement of the PCC/precuneus in AD [46, 54, 56]. In healthy individuals, the PCC/precuneus are characterized by higher rates of metabolism and cerebral blood flow than those of the global brain mean [57]. In humans, the PCC/precuneus develop phylogenetically and ontogenetically late, making them the last to become myelinated and the first to be affected by pathological processes [46]. Because of their thinner myelin sheaths as well as unique metabolism, connectivity, and vascular characteristics, the PCC/precuneus are preferably vulnerable for neurodegenerative processes, such as amyloid deposition, which are observed in the early stages of AD development [46, 57–59]. In terms of AD genesis, previous studies have focused on the MTL; however, increasing attention has shifted to the PCC/precuneus because of their key role in disrupting memory during initial AD development [46]. Compared to structural MRI and cerebrospinal fluid (CSF) tau biomarkers, FDG-PET abnormalities in the PCC/precuneus provide the strongest, earliest (preceding cognitive impairments) indication of individuals who will later progress to AD [58]. Our meta-analysis supports this evidence and consistently identified regional hypometabolism at baseline in the PCC/precuneus as an effective predictor of aMCI to AD conversion. Progressive dysfunction of the PCC/precuneus might lead to impaired neural connectivity and vice versa, revealing the neurodegenerative process that ultimately results in cognitive dysfunction and clinical symptoms. Thus, regional hypometabolism in the PCC/precuneus appears to be an imaging marker that can be used to characterize AD development.

Interestingly, in terms of consistency of prediction, we observed an asymmetric pattern of regional hypometabolism. Although in the main voxel-wise meta-analysis, the right PCC/precuneus was identified, these regions were associated with significant between-study heterogeneity. Including more eligible studies in future investigations may increase our statistical power. The underlying neurobiology of the asymmetric involvement remains unclear; however, increasing evidence indicates greater susceptibility of the left hemisphere to degeneration than the right. Moreover, it has been suggested that brain structures located in the left hemisphere are affected earlier and more severely in AD [60–65]. Indeed, greater metabolic dysfunction has been reported in the left hemisphere when compared to that of the right in patients with AD [61]. Further, a meta-analysis of voxel-based morphometry studies conducted by Ferreira and colleagues found that atrophy of the left MTL was the most robust brain structural biomarker in predicting conversion from aMCI to AD [66]. The PCC/precuneus have intimate functional and structural connections with the MTL, which collaborate the accordance of lateralized alterations described by Ferreira et al. Moreover, previous studies have demonstrated dynamic alterations in the degree of left-right asymmetry during disease progression [63, 67–69]. Consequently, a left hemispheric predominance of regional hypometabolism and atrophy in these regions might be another early characteristic that could be used to track disease progression.

The findings of the present meta-analysis were not consistent with those of a prior meta-analysis that combined three different imaging modalities (PET, SPECT, and structural MRI) [24]. That meta-analysis identified the left inferior parietal lobe and right precuneus as the brain regions most predictive in differentiating MCI converters from non-converters [24]. This discrepancy is not surprising and can be explained by the several factors. First, the results of the previous meta-analysis are hard to interpret and may be biased due to their use of several analytic approaches and modalities, which represent different neuropathophysiological information [24]. In contrast, the findings of our study are easier to interpret and understand as we only included FDG-PET studies that provide unique metabolic alterations. Second, we used a SDM approach in the current study, which is based on and modifies previous meta-analytic methods, (e.g., the ALE and multilevel kernel density analysis) [29, 70–72]. Specifically, the SDM introduces a series of improvements and novel features, allowing us to further conduct comprehensive complementary evaluations, including sensitivity, heterogeneity, publication bias, and meta-regression analyses, to test the robustness of our findings and to minimize the risk of false positive results [26, 28–30, 70–72]. Third, the current meta-analysis included eight more published datasets, which increased our statistical power. Together, these factors make the current results more accurate than the previously published meta-analysis.

In addition to the left PCC/precuneus identified in the main voxel-wise meta-analysis, several other brain regions exhibited hypometabolism, such as the right PCC/precuneus, bilateral ACC, and left middle temporal and frontal gyri. However, the finding of altered glucose metabolism in these regions was not very reliable, as determined by additional complementary analyses of jackknife sensitivity, heterogeneity, publication bias, and meta-regression. Several moderator variables were examined to understand the potential source of this heterogeneity. The meta-regression analyses indicated that mean age and severity of cognitive impairment at baseline as well as follow-up duration had significant effects on alterations in brain metabolism. Many other moderators, such as age at onset, disease duration, education level, aMCI subtype (amnestic single domain and multiple domains), genotype, and cerebrovascular risk factors might also affect brain metabolism. In addition, variations in imaging protocols and analytic procedures across studies may have influenced the observed heterogeneity. However, we were unable to address many of the described confounding factors as we were limited to performing analyses on data available from the included studies. Future studies with large, homogeneous samples and standardized protocols are warranted to control these moderator effects and to verify the results of our meta-analysis.

Although our comprehensively modified meta-analysis enabled us to successfully identify which brain regions might serve as predictive indicators of conversion from aMCI to AD, the present study had several limitations. First, the SDM technique was based on reported peak coordinates and their effect sizes rather than raw imaging data. Although this is common practice in coordinate-based meta-analyses, the approach may produce less accurate results [26, 28–30, 70–72]. Future studies based on statistical parametric maps or raw imaging data would highly increase the power of our results [73, 74]. Second, the exclusion of studies that did not report stereotaxic coordinates likely reduced our power to detect less-robust alterations, and may have biased our findings [75]. Third, the results identified in our meta-analysis were based on between-group FDG-PET differences. Further investigations would benefit from utilizing these results as selected reference ROIs for discrimination on an individual basis. Fourth, the present meta-analysis only included FDG-PET studies. The use of FDG-PET, in combination with other imaging and CSF evaluation approaches, such as resting-state functional MRI, structural MRI, tau and perfusion imaging techniques, as well as CSF β-amyloid and tau level assessments, should be advocated as this integrated information would increase the power for early AD detection [58].

## Conclusions

Using a modified meta-analytic approach, the present study demonstrates the most robust FDG-PET changes in the left PCC/precuneus in aMCI converters relative to aMCI non-converters at baseline. This finding has important implications in understanding the neural substrates for the prediction of conversion from aMCI to AD. Hypometabolism in the left PCC/precuneus was determined as an early feature in the development of AD and this might serve as a candidate imaging biomarker for early detection and for tracing disease progression. Further work is warranted to determine the potential of these findings in clinical use and their value in the development of future therapeutic interventions in individuals at the predementia stage of AD.

## Additional files

Additional file 1 **Figure S1.** Regions of the heterogeneity map from the SDM heterogeneity analysis. Abbreviations: SDM, Seed-based *d* Mapping; L, left; R, right. The color bar indicates the maximum and the minimum SDM-Z values. (TIFF 684 kb)

Additional file 2 **Figure S2.** Funnel plots of the peak coordinates from the main meta-analysis for detecting publication bias. Abbreviations: MNI, Montreal Neurological Institute. (TIFF 184 kb)

## Abbreviations

ACC: Anterior cingulate cortex
AD: Alzheimer’s dementia
ALE: Anatomical likelihood estimation
aMCI: amnestic mild cognitive impairment
BAs: Brodmann areas
FDG-PET: ^18^F-fluorodeoxyglucose positron emission tomography
FWHM: Full-width at half maximum
MMSE: Mini-mental state examination
MNI: Montreal Neurological Institute
MOOSE: Meta-analysis of observational studies in epidemiology
MRI: Magnetic resonance imaging
PCC: Posterior cingulate cortex
ROI: Regions of interest
SDM: Seed-based *d* Mapping
SPECT: Single photon emission computed tomography
