# Supplementary figures and images for: Cerebral glucose metabolic prediction from amnestic mild cognitive impairment to Alzheimer’s dementia: a meta-analysis
Source: Transl Neurodegener. 2018 Apr 23;7:9. doi: 10.1186/s40035-018-0114-z (PMC5911957; doi:10.1186/s40035-018-0114-z)

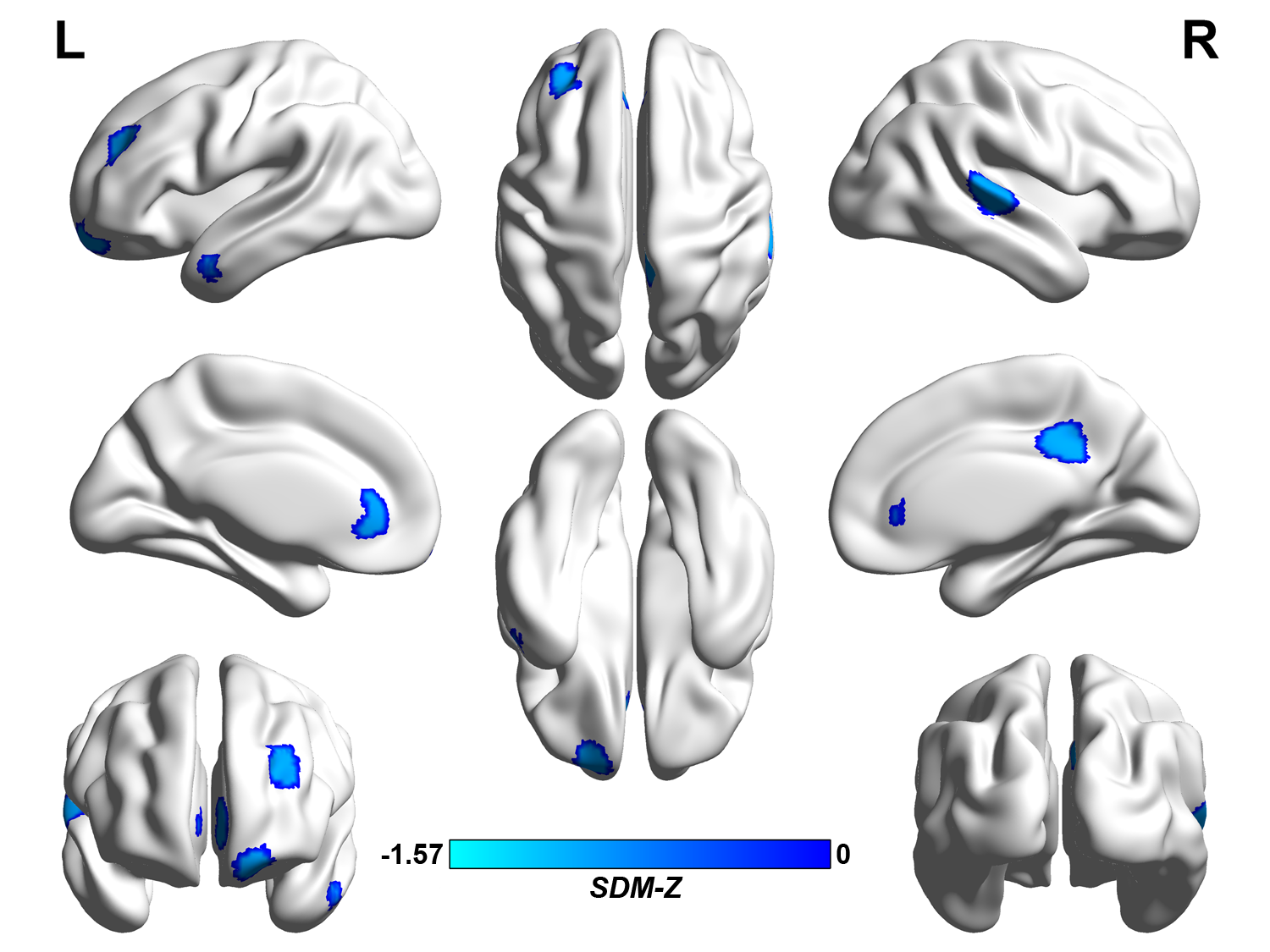

Supplement: Supplementary file 1 — Figure S1. Regions of the heterogeneity map from the SDM heterogeneity analysis. Abbreviations: SDM, Seed-based d Mapping; L, left; R, right. The color bar indicates the maximum and the minimum SDM-Z values. (TIFF 684 kb) [file 40035_2018_114_MOESM1_ESM.tif]

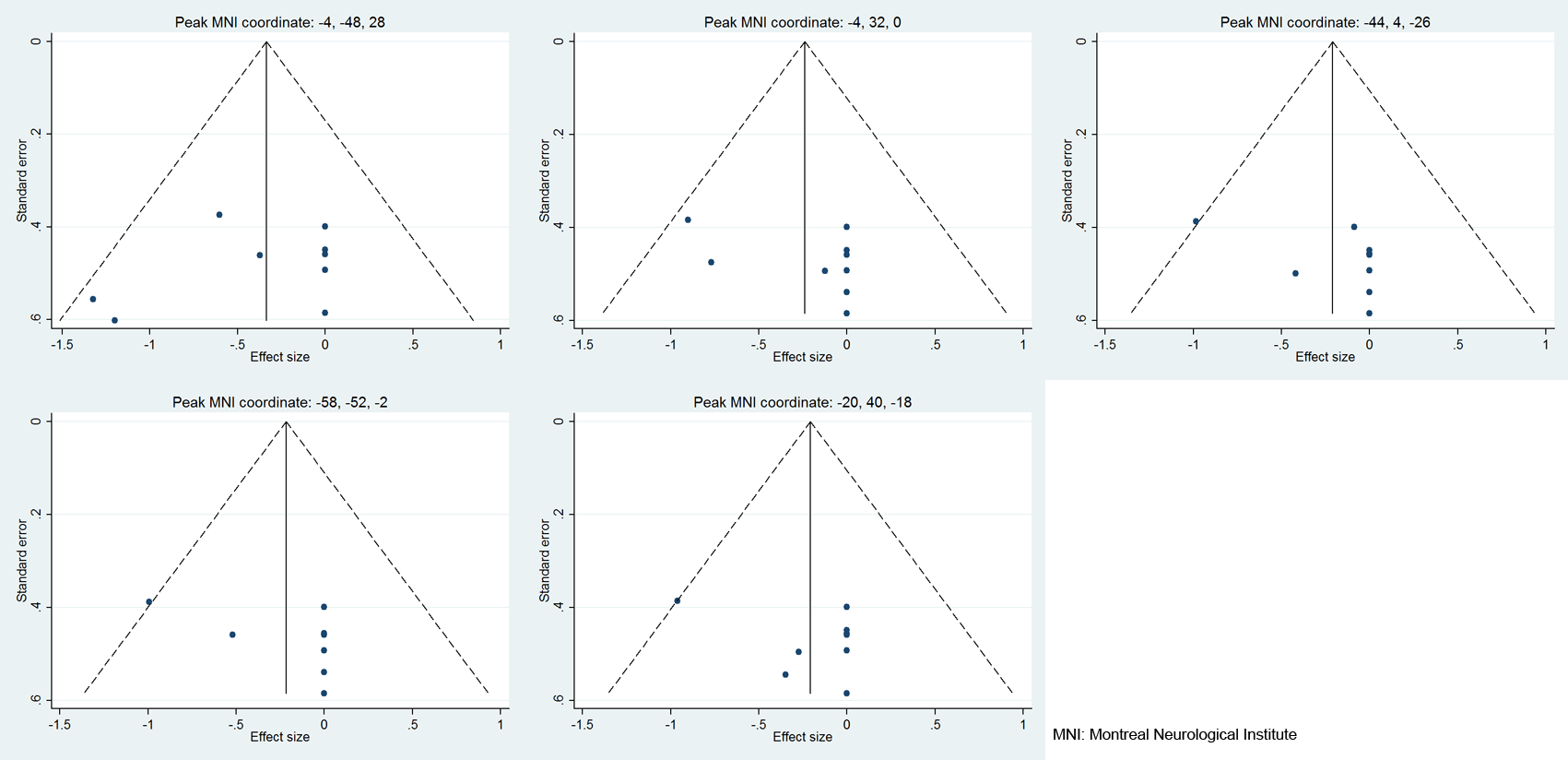

Supplement: Supplementary file 2 — Figure S2. Funnel plots of the peak coordinates from the main meta-analysis for detecting publication bias. Abbreviations: MNI, Montreal Neurological Institute. (TIFF 184 kb) [file 40035_2018_114_MOESM2_ESM.tif]
